# Professional Excellence

**Published:** 1874-03

**Authors:** W. W. H. Thackston


### AMERICAN JOURNAL

OF

### DENTAL SCIENCE.

**Vol. VII. THIRD SERIES--MARCH, 1874. No. 11.**

### ARTICLE I.

### *Professional Excellence.*

An Address of Dr. W. W. H. Thackston, President of the Virginia State Dental Association—delivered at its last Annual Meeting.

*Gentlemen of the Virginia Dental Association :—*

It is cause of grateful acknowledgment to that Being whose eye is upon us all, and whose will controls the destiny of men and associations of men, that we are permitted once more to assemble in our corporate capacity to promote the ends, and cultivate the growth and improvement of a necessary and benign calling; to make another step in that progress which marks the age and distinguishes the other pursuits and professions of life. And especially should we recognize that mercy and benevolence which has watched over and preserved our lives amid the casualties and perils that beset us; and amid "the pestilence that walketh in darkness, and destroyeth at noontide." So far as we are informed, none of our members have fallen since our last annual assemblage, but all live to testify the infinite love and forbearance of that Sovereign whose creatures we are, and who holds our breath in the hollow of His hand.

It is also a peculiar satisfaction to witness the evidence of increased interest in the objects of our association, manifested by this presence. We see around us some of the old and honored representatives of our profession; the fathers of Virginia dentistry, the pioneers, guides and leaders, who struck the rock when we were in the wilderness of darkness and empiricism; who lifted up the rod, that the faint might look and again be strong; and who have at last brought us to a view of that promised land, where true science reveals a prospect enchanting as that which filled the eyes and gladdened the hearts of Israel's sons and daughters. We find here brethren in the prime and strength of manhood, with their faculties matured, their judgments ripened, and their confidence unshaken in the brilliant promise which awaits, and will reward our earnest and faithful labors; and here too, are our younger brothers with the generous impulses, the ardent enthusiasm and noble aspirations, which stir and animate our hearts, and nerve and strengthen our arms in every good word and work. It is no trivial subject or unimportant matter which thus attracts the presence, and commands the attention of such a body of men; and we again repeat the expression of our profound satisfaction, that we have assembled with unbroken ranks, and under auspices so favorable; and again may we repeat the acknowledgment of our gratitude to the Great Disposer of events, and invoke His guidance and blessing upon our transactions. And now, gentlemen, we confess we have been not a little embarrassed in selecting a suitable and appropriate subject for our annual address; and have with doubt and hesitancy chosen *Professional Excellence* as perhaps the best we could present for your entertainment and instruction.

As a general proposition, professional excellence implies a preparation, as thorough and complete as practicable, for the duties and offices of our chosen calling. As a profession, dental surgery is no longer an open domain, and the day has passed when men might somersault from the varied and incompatible pursuits of life, and appear full-fledged professors and practitioners in our specialty of the healing art.

I shall not consume your time, or weary your patience with a portraiture of the "dark age" of dentistry, an age sombre and melancholy to contemplate; but here and there, relieved by some of the brightest examples of excellence that any department of science can boast; examples of genius, of energy, of patient, persistent application and research, which have written their names in imperishable characters in the annals of our craft, and built for them monuments as enduring as the records of science. Fouchard, the father of modern dentistry, Dellabarre, Desirabode, Bourdette, Bunon, Tenon, Le Foulon, Bichat and Blandin, of France; Hunter, Bell, Blake and Fox, of England; Greenwood, Hudson, Hayden and Gardette, of America, are all names which the dentist of to-day, and the dentist of the future, may safely accept as examples of an excellence most rarely attained. These men were all contemporaneous with the embryonic age of modern dentistry,—beyond a few suggestions and aphorisms of Hippocrates, a few allusions of the Latin poets, a few observations of Galen, and a more extended and respectable essay of the great old French surgeon, Ambrose Pare, nothing was known of the anatomy, physiology, pathology, or therapeutical needs of the teeth. There were no accepted theories, and no recognized systems or principles of practice; and yet, from the individual and almost unaided efforts of these men, system was brought out of confusion, and such light eliminated from the surrounding darkness, as to create the dawn of that day, in which it is our fortune to live. These were our early illustrators of excellence, who prepared the way, and heralded the advent of such teachers as Nasmyth, Owens, Goodsir, Richardson, Tomes, Beale, Arnold, Huxley, and others of England; of Raschkow, Retzius, Parkinge, Kolliker, Leber, Rottenstein and Wedl, of Germany; of Hayden, Harris, Arthur, Taft, Richardson, Piggot, Handy, Bond, and many others of this country, who have distinguished themselves as writers and operators. And in passing, we may remark the singular and inexplicable fact, that during the last twenty or thirty years, France, the fatherland and birth-place of modern dentistry, has produced no distinguished writer or practitioner in our specialty; its very court and nobility being dependent upon American dentists for the services they require.

But to proceed with our subject. We have said that professional excellence demands as a primary condition, adequate preparation; and more than this, it requires a personal and individual adaptation to the peculiar nature of our calling. It may be accepted as an axiom, that relatively few men possess the natural endowments essential to excellence in any of the departments of surgery; as relatively few exhibit the attributes which constitute the poet or the painter. These qualities and characteristics, no amount of training, no degree of application, and no known system of education can ever secure; they may be cultivated and developed, but *never created* or *acquired*; and yet, they are indispensable to respectability, not to say excellence in all surgical practice.

A pure taste, a correct eye, a well adjusted nervous and muscular organization, a facile and cunning hand, which may be steady, firm and strong, or delicate and tender in its touch as the breath of a vernal morn, and an inventive faculty that will at once respond to all the exigencies and ever changing conditions of cases in practice, are some of the distinctive qualities of the dentist who may aspire to excellence in his profession; and no man wanting in these natural endowments should be encouraged to adopt any department of surgery, and least of all dental surgery as a profession. It is a fact, attested by observation, and confirmed by all experience, that while most men of average intellectual endowments and educational advantages, may make of themselves safe and efficient practitioners of medicine, it is only here and there that you find one who attains respectability or distinction as an operative surgeon. The explanation is simple and manifest. The one possesses the requisite *natural qualities*, the other, wanting in these, can no more be educated into them, or have them educated into *him*, than you can educate the average man into the sculptor or the orator; and I repeat, that it is always a personal and professional misfortune that such should ever adopt dentistry as the business of life.

The dentist then must be a man of natural mechanical aptitude, of manipulative dexterity, and of artistic eye and correct taste. The requirements for the cultivation and development of these qualities, as well as for the anatomical, physiological and other departments of professional education *are*, or *should* now be fully met in the offices and laboratories of established practitioners, in the standard text books now so amply provided, and in the lecture halls, laboratories and infirmaries of the colleges. We can suggest no improvement in the present system of professional education if properly carried out, and faithfully illustrated by our college faculties; it is true, that expressions of dissatisfaction have lately been heard concerning the management of our schools, and the conduct of our "faculties of instruction." There may be faults and delinquencies, and there *are* imperfections which are common to all human enterprises that attach to dental colleges, but it cannot be denied that they are institutions of inestimable value, that they have done more than all other agencies combined, to give to dental surgery its present status among the liberal professions and its present consideration and esteem in society. And further, it cannot be denied or gainsaid that our Professors and college faculties have been generous, laborious and self-sacrificing, and as a general thing are an unrequited and uncompensated set of men. Whatever faults or imperfections may now challenge our criticism, dental surgery owes a debt of gratitude to these institutions which has never yet been paid. The period of pupilage may be too short—we think it is; the standard of proficiency for graduation may be too low; the struggle for sustaining patronage

(owing to an unfortunate competition, incident to the unwise multiplication of colleges,) may have given ground for some of the strictures and animadversions these schools have received. But admitting all that has been charged, it is simply an *abuse*, which in nowise touches the principles, or impairs the system upon which these institutions were founded; and we may rest assured that whatever errors or imperfections may now exist, will be speedily corrected by a wholesome professional sentiment, which no school can resist and live. Our colleges *must* meet the demands of the age, or go down and be succeeded by others in harmony with the times, and measuring up to all the duties and responsibilities of advanced and advancing science.

Dental surgery has made in the last thirty years most wonderful strides,—in three decades, more has been accomplished than in all the preceding *centuries*, from *Hipocrates* and *Galen* down to the days of Greenwood, Hudson, Harris' and Hayden ; so that we do not perceive the necessity, or recognize the advantages likely to result from a change, or from any radical modification of our present system of professional training. But highly as I appreciate our educational facilities, and earnestly as I would commend the advantages they present, I am proud to assert, that some of the brightest exemplars of excellence in dentistry, achieved that excellence and distinction without such aids and helps. Hudson, Hayden, Parmley, Roper, Gardette and Maynard, with others who might be mentioned, wrought for themselves imperishable names through their individual and almost unaided labors ; neither of these men, so far as we are informed, ever having matriculated in any other than academic schools ; and I have the honor and satisfaction of addressing members of the Virginia Dental Association, who have without the help of colleges or Professors, earned by the force of genius, and the exhibition of personal energy and determination, positions of eminence and awards of distinction, which the proudest may court and the more favored may never reach or attain.

But, complete and thorough as may be our preparation for dental practice, fortunate as may have been our circumstances and surroundings in this regard, we have simply laid the foundation upon which we may subsequently build an enduring monument of character and reputation; we have but learned the alphabet of that language which, by diligent study and cultivation, will unfold to us the beauties, and unlock the treasures of that excellence for which we would have you strive. A determined purpose to faithfully use the knowledge and employ the resources we have already acquired, to increase that knowledge and multiply those reresources, must be our animating spirit and controlling desire. Keep abreast with the advance of science, read carefully the current literature of the profession, study closely the new standard works as they appear, investigate the theories, and analyze the systems of practice as they are introduced, appropriate and make our own every improvement and every valuable discovery, and make them all the better and more valuable to humanity for having passed through our heads and hands. Accept freely, and give generously all that we, or our fellow laborers in the field may invent, discover or create. Never permit our interest to abate, or our efforts to relax while a single theoretical problem remains unsolved, or a single practical question is undecided. It is the saddest era in the life of a professional man, when he concludes that he has nothing more to learn; that he has mastered all the requirements of his calling, and compassed all the resources of his vocation. With *that* moment begins his decline in progress and usefulness, and *with* that moment ends all rational hope of future excellence or personal distinction.

Why fellow members of the Virginia Dental Association, the most familiar expression of disease which the dentist is required to combat—caries of the teeth—is even now enshrouded in mist, and fog, and uncertainty as to its cause, progress and final result. Who can with certainty tell us after a century of research, aided by all the appliances of modern science, the *exact* cause or causes of decay of the human teeth? We are familiar with the various theories which have from time to time formed the basis of practice with our best men; but we have seen these theories one after another fade out, melt away, become modified by new revelations, or utterly and wholly repudiated in the light of subsequent experience and observations. The chief duty and most important office of the dentist is to *save* the natural teeth, and if we knew *exactly* what caused decay and destruction of these organs, and precisely and certainly how that cause operated in all cases, with our present knowledge and resources, it would be a *short* step to discover the means of counteracting that cause, and preventing its ravages in dental tissue. But with all the labor and all the research, with all the aids of advanced physiology, of analytical and synthetical chemistry, with all the wonderful revelations of the microscope, the cause and methods, so to speak, of decay of the natural teeth remains an undetermined and perplexing question. One will tell you that this cause is local; another, that it is general or systematic; one, that it is external; another, that it is internal; one, that it is mechanical; another, that it is chemical; and still another, and we think a more rational theory is, that it is chemico-vital; one, that it is acids; another, that it is alkalies in the oral and buccal secretions, and more recently, the disclosures of the microscope would seem to warrant the conclusion, that animal and vegetable parasites, animalculæ and infusoria, play a very active and decided part in the process of dental disorganization and decay. I merely mention this single example, lying upon the very threshold of our specialty of science, as an illustration of the necessity for continued and persevering effort on the part of all who would achieve excellence as dental surgeons; and the stupidity and folly of any man in our profession who would persuade himself that he knows enough to cease his labors, and rest upon his present attainments. But the timid and the weary ask, what chance is there for us? What need of our worrying our brains with that which has baffled the efforts, and defied the research of scientists for a century or more? Why, simply this; that every step is an approach to truth, though in itself a failure, though disappointment may result, and though we may at times appear in a certain sense to be wandering away from the object sought; in another, and *stronger sense*, every additional step is an approach to the desired end, upon the principle or the philosophy of the hunter, whose flagging companion, after beating the fields and finding no game, urged the abandonment of the hunt. "No," said the keen old sportsman, "I know there *are* birds; we *now* know they are not in these fields; let us look elsewhere, and we will find them." We know that human teeth deeay, and myriads of these invaluable organs are annually lost, we have beaten many fields in the vain pursuit of the cause of that decay and loss; let us now look elsewhere, and continue the search till success rewards our diligence and perseverance, and we finally reach a solution of this great and important problem.

In this connection I would earnestly commend the value and importance of physiological study and investigation and of histological research and examination; for while no absolutely certain or definite results have yet been reached through these agencies, yet, from the startling revelations which have rewarded the labors of the physiologist, the chemist, and the histological anatomist, we are led to the reasonable hope, that through the microscope, through the crucibles and retorts of the laboratory, and the persevering investigation of the elements and germs of matter, and the combination and development of those elements and germs into tissues and organs, perfect in *form*, *composition* and *functions*, that we *may* at no distant day have unfolded to our view, not only the secrets of vital organization, but the true and complete explanation of disorganization and destruction of the special organs of the human body, committed to our care and preservation; and just here, I would bring to your notice, and commend to your earnest attention, "Richardson's Hand Book of Medical Microscopy," and

"Tyson's Cell Doctrine," a work of great interest and value to the dentist, for it is really what its title indicates, "a *resume* of all that has been written by Virchow, Robin, Huxley, Hughes, Bennett, Beal, and other distinguished microscopists and physiologists." Tyson has also recently published a small work, "an introduction to the study of practical histology for beginners in microscopy," that is of great value to dentists who are new to this subject, and who are unfamiliar with the qualities and practical uses of the microscope.

If we would attain excellence, I repeat, we must be diligent students, patient investigators, and accurate observers, so long as we assume the responsibilities, and essay the duties and offices of special surgeons. Our current literature should be closely scanned, and our standard works, both American and Foreign, should receive our critical attention; and our own discoveries, inventions and improvements, should be carefully noted, and generously given to the cause of science and the interests of humanity.

[TO BE CONTINUED.]

---

